# A 2.5-Year Weight Management Program Using Noom Health: Protocol for a Randomized Controlled Trial

**DOI:** 10.2196/37541

**Published:** 2022-08-12

**Authors:** Robyn Sysko, Jessica Bibeau, Allison Boyar, Kayla Costello, Andreas Michaelides, Ellen Siobhan Mitchell, Annabel Susanin, Tom Hildebrandt

**Affiliations:** 1 Eating and Weight Disorders Program Icahn School of Medicine at Mount Sinai New York, NY United States; 2 Noom Inc New York, NY United States

**Keywords:** weight loss, weight loss maintenance, digital health, Noom, Diabetes Prevention Program, DPP, mobile phone

## Abstract

**Background:**

Overweight and obesity are serious public health concerns. As the prevalence of excess weight among individuals continues to increase, there is a parallel need for inexpensive, highly accessible, and evidence-based weight loss programs.

**Objective:**

This weight loss trial will aim to examine the efficacy of the Noom weight loss program in comparison to a digital control after a 6-month intervention phase and a 24-month maintenance phase, with assessments continuing for 2 years beyond the intervention (to 30 months—after the baseline). The secondary outcomes include quality of life, psychosocial functioning, sleep quality, physical activity, diet, and health status. This trial will also examine the severity of obesity-related functional impairment, weight loss history, and demographic moderators, along with adherence and self-efficacy as mediators of the outcome.

**Methods:**

A total of 600 participants were randomized in a parallel-group, controlled trial to either Noom Healthy Weight Program (intervention) or Noom Healthy Weight Control (control) for a 6-month intervention. Both intervention and control groups include diet and exercise recommendations, educational content, daily logging capabilities, and daily weigh-in entries. The Noom Healthy Weight Program also includes a coach support for weight loss. Remote follow-up assessments of eating, physical activity, psychosocial factors, app use data, and weight will be conducted at 1, 4, 6, 12, 18, 24, and 30 months after baseline. Weight is measured at each follow-up point during a Zoom call using the participants’ scales.

**Results:**

Enrollment began in March 2021 and the 6-month intervention phase ended in March 2022. Data collection for the final assessment will be completed in March 2024.

**Conclusions:**

This study tests commercially available digital lifestyle interventions for individuals with overweight and obesity seeking weight loss support. Data obtained from the study will evaluate whether the Noom Healthy Weight Control Program can help individuals overcome weight loss, achieve long-term maintenance, adhere to lifestyle changes, and feature use barriers that are present in other traditional weight loss treatments.

**Trial Registration:**

ClinicalTrials.gov NCT04797169; https://clinicaltrials.gov/ct2/show/NCT04797169

**International Registered Report Identifier (IRRID):**

DERR1-10.2196/37541

## Introduction

Overweight and obesity have reached epidemic proportions in the United States [1], with prevalence rates reaching 42% between 2017 and 2018 [2]. Excess weight represents a serious public health concern because of the increased risk of medical sequelae (eg, high blood pressure, sleep apnea, or COVID-19) [3] that require lasting and expensive interventions. In-person behavioral treatments, such as the Diabetes Prevention Program (DPP) [4], are generally effective, producing 4% to 8.3% weight loss after 6 months [5,6], 3.9% to 8.6% after 12 months [6,7], and similar or worse outcomes compared with web-based deliveries [6]. However, weight loss with these interventions is not always sustained over time, weight regain after treatment is common [8,9], and in-person services can be prohibitive with regard to cost and accessibility [6,10,11], as DPP treatment costs more than medication treatment alone (>US $1300 more per participant) in the first year of treatment [10]. Therefore, web-based delivery may improve accessibility and provide a more cost-effective option for in-person weight loss treatment without compromising success [6]. Thus, a major challenge in using behavioral weight loss interventions more broadly is scaling extant empirically based strategies while simultaneously optimizing personalized elements that may help maintain weight loss and lifestyle changes over time. Programs adapted to deliver behavioral weight loss via a mobile app could help overcome these limitations [12].

With approximately 97% of Americans owning a smartphone [13], digital health resources are becoming increasingly accessible [14], and ≥37,000 health or weight apps were available as of 2019 [15]. However, many apps lack evidence-based strategies used in face-to-face behavioral treatments, such as coaching and personalized feedback [16], and provide only self-monitoring and goal-setting [17]. When strategies such as problem solving [18], coaching [19], or increased social support [20,21] are included as features, the observed weight loss results prove significant [22]. Specifically, a pilot project found significant weight loss (1.6% and 2.3%) using problem-solving strategies in a weight loss app over 8 weeks [18]. A program that included social support reported significant group differences in weight (−5.3 kg) compared with a control (−2.2 kg) [21]. Finally, when coaching was added to a weight loss maintenance program, the coaching groups did not show weight regain, whereas there was significant regain in the noncoaching group [19]. Weight loss maintenance is limited; however, only 20.6% of people sustain decreases for a year [23], and approximately 70% of weight loss is regained within the first 2 years [24]. While digital weight loss programs can be successful [11,22,25-28], improvements are needed [16], including a platform that provides comprehensive intervention focusing on both short-term weight loss and long-term maintenance.

In 2008, Noom Inc created a mobile intervention modeled on strategies from the DPP with a goal of improving the accessibility of personalized expert guidance [29,30]. Noom Inc focuses on healthy behavior changes through manageable goal setting, daily monitoring, coach feedback, and a social support group [29,30]. In comparison to other apps for weight loss, the Noom program includes a large proportion of behavioral strategies derived from an evidence-based weight loss program (ie, DPP), including a physical activity goal, exercise safety (both compared with 20% of other apps), benefits of a healthy lifestyle (13.3% of other apps), food substitutions (10% of other apps), stimulus control, portion control, lifestyle activity (6.7% of other apps), problem solving (3.3% of other apps), stress reduction, relapse prevention, negative thinking, social cues, regular eating patterns, and time management (0% of other apps) [31]. Furthermore, only Noom and another app were observed to incorporate all assessed core and motivational interviewing constructs [32]. Over 6 months, 77.9% of Noom users in the general population reported weight loss [33], and those demonstrating greater adherence reported better outcomes [34]. The study described below will extend prior studies of Noom Inc weight loss products using a randomized controlled design with a large sample to test the efficacy of the Noom Healthy Weight Program (intervention) versus Noom Healthy Weight Control (control), during a 6-month intervention and 2-year maintenance phase. In addition, the primary difference between the intervention, which is a commercially available Noom product, and the control, a version created specifically for this research study, is the inclusion of coaching. Thus, this project will help evaluate whether coaching is an essential element in the effectiveness of the intervention. Furthermore, prior studies examining the efficacy of a Noom Inc product were either uncontrolled, did not include long-term follow-up, or failed to include an active digital control group [33,34]. This project will therefore improve extant research by incorporating coaching, problem solving, social support, and a digital control group, and extending the assessments over 30 months, allowing a 24-month follow-up assessment period.

Moderators (or variables that potentially alter the effect of treatment on outcome) are often difficult to replicate in behavioral interventions and a host of demographic, sociocultural, and comorbid health conditions that offer a proxy for obstacles to acceptance, engagement, and outcome [35]. We will use a composite moderator approach to capitalize on the aggregation of potentially small but clinically meaningful effects [36]. Our proposed moderators will include age, comorbid health conditions, demographics (sex, marital status, race or ethnicity, education, and socioeconomic status), and psychosocial functioning. There are well-documented moderating effects of age (favoring younger individuals) on the acceptance and use of mobile health platforms [37] and evidence that social determinants, including sexual identity, socioeconomics, and education, are theoretically robust contributors to digital health engagement, but understudied in the published literature. These individual aspects of identity, health, and status are likely to affect expectations regarding the value of digital interventions, their applicability to individual circumstances, and the acceptance of specific tools used within the program.

Weight suppression, or the difference between current and highest weight, has demonstrated predictive effects for obesity-related prevention [38] and evidence of attenuated weight loss in behavioral weight loss interventions [39,40]. These moderating effects are thought to occur because of the psychological impact of deprivation and physiological adaptations related to weight suppression. We predicted, in line with others, that those with greater weight suppression at baseline would lose less weight and be more likely to regain weight during the follow-up period. This hypothesis is based on the finding by Wing et al [41] that weight suppression leads to larger time-dependent weight regain in the context of obesity prevention.

**Figure 1:**
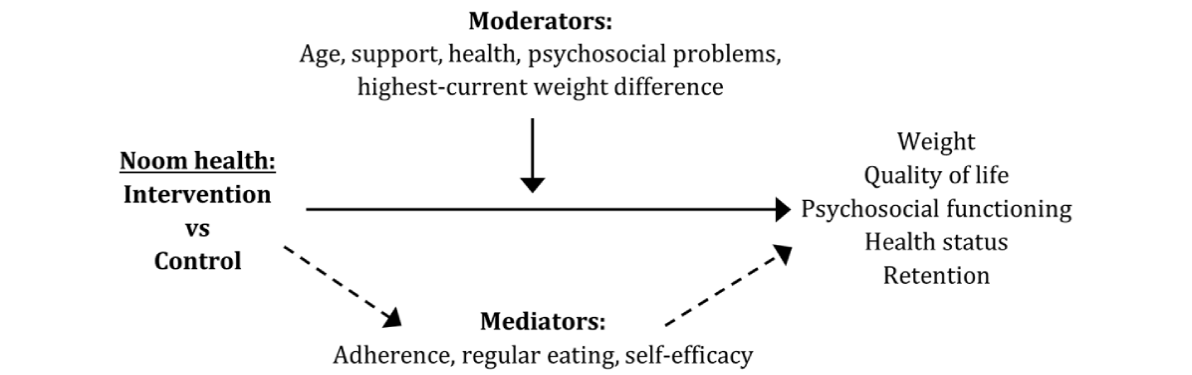
Hypothesized moderator and mediator effects.

Little is known about how digital program features account for efficacy or long-term outcomes [12]; however, there is evidence that personal communication [2,18,19] and self-monitoring mediate weight loss and long-term outcomes [27,30,33,42,43]. These mediator effects largely depend on the evidence of treatment engagement (adherence to recommendations, use of digital tools, and participation in digitally delivered interactions). Consequently, we tested a time-series model that examined the lagged effects of these engagement elements on attrition, weight, and psychosocial outcomes. In addition, we tested whether self-efficacy and regular eating mediate these outcomes. Self-efficacy, which is directly targeted by the Noom intervention, has demonstrated mediation effects in weight loss and sustained weight—after the intervention [44], and regular eating, when experimentally manipulated, is associated with greater reductions in energy intake and associated weight loss [45]. Consequently, we hypothesized that both self-efficacy and regular eating would mediate weight loss at the end of the treatment and follow-up. Figure 1 displays the hypothesized moderator and mediator effects tested in this study.

## Methods

### Study Overview

A total of 600 participants were randomized to receive either the Noom Healthy Weight Program (intervention) or Noom Healthy Weight Control (control), delivered in a smartphone app format. The Noom Healthy Weight Program includes diet and exercise recommendations, communication from a coach to help with goal setting and barriers, dietary logging, daily weigh-ins, articles, and access to the social community on Noom. Noom Healthy Weight Control includes daily logging and the same recommendations, but communication with the coach and social interactions are not available. The primary intervention period occurred during months 1 to 6 and the maintenance phase during months 7 to 30. Follow-up assessments will be conducted at 1, 4, 6, 12, 18, 24, and 30 months after the baseline. Additional details regarding these elements are provided in the following sections.

The project was funded by Noom Inc with participants recruited across the United States who contacted the company expressing interest in their research, which should allow for greater generalizability of app users seeking digital weight loss advice. All study procedures were remote and were conducted by the Icahn School of Medicine at Mount Sinai Eating and Weight Disorders Program.

### Ethics Approval

The protocol for this trial was reviewed and approved by the Mount Sinai Institutional Review Board (STUDY-20-01299M).

### Study Aims

The primary aim of this study is to test the comparative efficacy of the Noom Healthy Weight Program (intervention) and Noom Healthy Weight Control (control) for weight loss at 6 and 30 months after baseline. The secondary aims are to test caloric intake, dietary behavior, physical activity, sleep impairment, quality of life, psychosocial functioning, and self-reported health status 30 months after baseline. It is hypothesized that greater improvement will be observed after the intervention in Noom Healthy Weight Program users.

Another secondary aim is to test the moderation of the treatment response. We hypothesize that a clinical profile determined via data reduction of relevant demographic and clinical features into a composite variable [35] will show a significant interaction by treatment condition, such that individuals with an increased risk profile (older age, less support, more health conditions, and psychosocial problems) will derive greater benefit from Noom Healthy Weight Program than Noom Healthy Weight Control. These indicators of the latent moderator potentially contribute small but meaningful moderation effects to the treatment. To increase power, they are combined into a latent variable, so the source variability associated with sociodemographic profiles and psychosocial symptoms (typically correlated in samples of individuals with obesity) will allow us to determine if the composite profile suggests a greater effect of the experimental intervention. Second, on the basis of the large extant literature on eating and weight disorders, we hypothesized that the difference between the highest weight and current weight will moderate the efficacy of the intervention compared with the control because it predicts outcomes for treatments that attempt to change eating behavior by imposing a restrictive structure (eg, traditional behavioral weight loss, regular eating in cognitive behavioral therapy for eating disorders, etc).

Exploratory aims include developing a dynamic model of adherence using a Bayesian belief network [46] that predicts individual changes in the primary outcomes with the intention of identifying within-treatment mechanisms of change in the Noom Healthy Weight Program. This model uses directed acyclic graphs to determine conditional relationships between global and local adherence. Global adherence markers must be present for local markers to occur (eg, app login is global adherence and enter weekly weight is local adherence). This initial model was then extended to a repeated-measures time-series model, resulting in dependent sequential adherence markers. A time-series model of adherence will be used to predict study dropout, successful weight loss, and quality of life. Adherence to intervention features and follow-up completion will be assessed at 12 months. In a separate model, we tested whether regular eating mediates the treatment effects of weight loss over time. We also expect that self-efficacy in weight loss will mediate the effects of the intervention versus control at and 12 months after baseline.

### Study Participants, Inclusion and Exclusion Criteria, and Recruitment Procedures

Eligible participants were between the ages of 18 and 60 years, expressed an interest in losing weight, were interested in using the Noom platform, had a BMI >27 kg/m^2^, and spoke English. Exclusion criteria included contraindications to smartphone use (eg, seizures from prior smartphone use), not owning a smartphone, acute suicide risk, current use of the Noom commercial program, and current or planned pregnancy over the next 12 months. Prior use of Noom products was not an exclusion criterion. Individuals who are pregnant should not participate in a medically unsupervised weight loss program.

Participants were recruited via links or advertisements on the Noom platform and website. Interested participants were directed to a link to complete an initial eligibility assessment on REDCap [47] and provided availability for a Health Insurance Portability and Accountability Act–compliant videoconference (Zoom) call with trained study staff, including the project manager, research coordinators, and students. During the Zoom call, participants completed the informed consent process, received a comprehensive description of the study, had the opportunity to ask questions, and provided their baseline weight. This discussion included an explanation of the importance of retaining participants in a research study, regardless of success with weight loss. In addition, participants were asked questions during the intervention and follow-up descriptions and study timeline to encourage discussion about possible barriers to participation and directly address any issues regarding time commitment. These procedures were conducted to help with retention, as outlined and discussed in a motivational interviewing approach geared toward enhanced retention [48]. A copy of the consent form was emailed to the participants before the call, and a signed copy was provided upon completion of the call. The participants then received instructions to complete the baseline measures (as described in subsequent sections) on REDCap. Participants received US $35 compensation to complete the baseline measures (approximately 60 minutes).

### Enrollment and Randomization Procedures

At this time, recruitment is complete, intervention and data collection are ongoing, and the analysis has not yet begun. After screening, eligible participants were randomized into intervention (n=300) and control (n=300) groups. Randomization was stratified by BMI and gender using R software (R: A Language and Environment for Statistical Computing; R Core Team) [49]. Randomization groups include 4 groups of BMI (27.0-29.9 kg/m^2^, 30.0-34.9 kg/m^2^, 35.0-39.9 kg/m^2^, and ≥40.0 kg/m^2^). Each BMI group was split into 3 sex categories (male, female, and other), creating 12 blocks. The randomization sequence created in R was programmed into the REDCap randomization module implemented by the project manager. This module allows for computer-automated randomization based on the defined parameters and concealment of the allocation order from all study staff and participants until after the intervention assignment. Only the project manager had access (concealed) to the randomization module to randomize the participants. After randomization, participants were notified of their assigned condition and information was sent to activate Noom.

### Noom Healthy Weight Program and Noom Healthy Weight Control

These interventions are provided at no cost to participants during this trial.

#### General Features of the Noom App

The Noom app interface includes (1) logging meals, weight, and physical activity, (2) reading curriculum material, (3) a recipe section to provide healthy meal suggestions, (4) talking to a coach, and (5) communicating with other group members for support. The group feature allows users to post personal challenges and successes, receive feedback from other users, and respond to others’ challenges. The participants were encouraged to use the group as much or as little as they wished. The coaches also participated in the group in response to posts and comments. Additional features of the app include a recipe section and the ability to log mood, sleep, glucose, and blood pressure.

#### Coach Role

Noom assigns users to a health coach who reviews participant entries in the app and works with participants to make changes in response to information entered in the app and communications between coach and participant via direct message. Weekly, the coaches reached out to users to help them identify individual goals, make and adhere to healthy changes, promote happiness and wellness, troubleshoot weight loss barriers, individualize feedback, find ways to adapt to lifestyle changes, and increase motivation [50]. Coaches used the chat feature to check in with participants through a private direct messaging system. They reached out to chat with each participant weekly to help set goals, resolve roadblocks, and provide support. Each week, coaches encouraged participants to establish weekly mini goals to help users reach their overall weight loss goal (eg, replacing afternoon snacks with a fruit or vegetable 5 days per week or going to the gym 4 times). After helping to establish goals, coaches used subsequent weekly contacts to check progress and set new goals. Coaches initiated conversations at the beginning of the intervention; however, participant-initiated contacts were welcome as well. There was no limit to the frequency of communication with the coach. The coach used motivational interviewing techniques during all conversations to establish actionable steps, set realistic expectations, encourage reflections, and identify the source of potential setbacks (eg, When was the last time you felt motivated? What has changed since then?). These conversations are geared toward promoting lifestyle changes aligned with the DPP. These techniques helped focus on communication by providing support, encouraging self-efficacy, promoting healthy changes, and helping to solve difficult situations.

#### Curriculum Content

The content provided during the intervention was based on the DPP and aimed to help users develop and maintain healthy eating patterns, increase physical activity, and foster skills to navigate their environment and overcome barriers. Topics include learning about calorie balance and the importance of sticking to daily calorie goals for weight loss, being active and finding time to be active daily, coping with situations or cues that create unhealthy behaviors, finding support in their environment from friends or family, and how to stay focused on adhering to changes [4]. Dietary changes include reducing fat and calorie intake, replacing snacks with healthy alternatives, and healthy choices while eating out [4], which is displayed in the logging feature of the app using a red light or green light system (ie, options with higher calories or fats are red). In addition, the content promotes other aspects of well-being, including regular eating, sleep (8 hours per night), and stress management. The intervention program delivers this content over the app in short, easily digestible daily material, and the control program provides this information over the app in weekly newsletters, with all content for that week provided in each newsletter.

#### Noom Healthy Weight Program

Participants are encouraged to use the features of the Noom app daily, including daily logging (diet, exercise, weight, mood, sleep, blood pressure, or glucose), reading daily curriculum material, viewing the recipe section, talking to a coach when applicable, and communicating with the group. As participants reach the 6-month mark of this intervention, there is a gradual transition from weight loss–focused content to maintaining weight loss, encouraging the diet and exercise changes that were made to become lifelong habits. Participants receive a one-to-one coaching call to evaluate the progress and re-establish goals for maintaining gains. In months 7 to 12, coaches continue to reach out once a week and conduct maintenance check-ins with participants to check the progress and resilience of changes (eg, brief and intense reinstatement of practices such as self-monitoring of food choices).

#### Noom Healthy Weight Control

Noom Healthy Weight Control is a version of the Noom program with limited features. This version includes the option to read the same curriculum material as the Healthy Weight Program, but presented in a weekly newsletter format, in addition to daily logging (same as the intervention, with the exception of mood and sleep logging) and the inclusion of a recipe section. The primary difference in this program is the lack of access to the coach or group, limited logging options, and the presentation of weekly curriculum materials instead of daily educational content. The maintenance phase for the control condition prompts users to continue to use exercise and dietary recommendations and to deliver new content based on these goals to keep users engaged.

### Treatment Fidelity

Coaches are trained in the areas of physical activity, nutrition, psychology, and behavioral health changes [50]. Coaches complete a health and wellness training program through Noomiversity, which teaches evidence-based strategies for weight loss and sustainability. Coaches complete an initial minimum level of training, including 75 hours of instructional content, mock scenario skill building, and 200 hours of direct user contact, and receive supervision from a research manager to ensure adherence to the intervention protocols. Research managers review coach transcripts every 4 to 6 weeks to provide guidance on nonadherence [50]. Coaches will be aware of users joining as part of this research project and will report any safety concerns to the study team. The user data will be shared with the investigators to perform adherence analyses.

### Assessments

Follow-up assessments will take place for all participants at months 1, 4, 6, 12, 18, 24, and 30 via a REDCap [47] survey link, and weight will be collected over Zoom using the participants’ scale. Participants will receive US $20 compensation for each follow-up assessment (approximately 60 minutes). The study team will monitor the adverse events that arise during these assessments. Should any conditions present harm to the participant if they continue with the intervention, they will either be withdrawn from the intervention or asked to take a break from the intervention until the condition subsides. These conditions will be assessed on a case-by-case basis, with the principal investigator (TH) making the final decision. All participants will be reached for follow-up unless they express directly to the study team that they would like to withdraw from the study and all future assessments. All adverse events, protocol deviations, and reportable information will be entered into the REDCap database.

#### Anthropometric Data

Participants will report their height at baseline and their weight at all assessment points. Considering that data collection occurs remotely, all participants will use their own scale to weigh themselves while on a Zoom call with a member of the research team, who will view the scale through the screen and record it in REDCap. Participants may schedule their Zoom call at any time of the day and wear any clothing that they prefer. There was no standardization of the scale used to measure the weight. The primary outcome of weigh-ins during assessments is to determine (1) the difference in weight from baseline to 6 months and (2) baseline to the 2-year follow-up, measured using BMI. The difference between the highest lifetime weight and current weight at baseline will be tested as a potential predictor of treatment response. Weight is collected at all assessment points.

#### Measures of Eating and Physical Activity

##### 24-Hour Recall Interview

The Automated Self-Administered 24-Hour (2020) [51], developed by the National Cancer Institute, is a self-administered dietary recall that evaluates foods and beverages consumed during a 24-hour period. Participants will complete one recall per assessment, which will be emailed to them during the baseline and follow-up windows for 8 administrations. The call can be completed on the day and time of the participants choosing. The difference in total caloric intake evaluated by the Automated Self-Administered 24-Hour from baseline to the 2-year follow-up is a secondary outcome.

##### Eating Disorder Examination Questionnaire

The Eating Disorder Examination Questionnaire [52] is a 28-item questionnaire assessing symptoms of eating disorder, with subscales for dietary restraint, eating, shape and weight concerns, and a global score. The difference in the global score from the baseline to the 2-year follow-up was a secondary outcome.

##### International Physical Activity Questionnaire

The International Physical Activity Questionnaire [53] is a 27-item self-report measure that evaluates the average time spent completing different levels of activity and generates a total activity in metabolic equivalents (MET) value for vigorous and moderate physical activity, walking, and sitting. The difference in METs from baseline to 2-year follow-up was a secondary outcome.

#### Psychosocial Measures

##### Depression Anxiety Stress Scales

The Depression Anxiety Stress Scales [54] is a 42-item self-report measure of negative emotional states with subscales for depression, anxiety, and stress symptoms. The difference in each of these subscales from baseline to the 2-year follow-up period was a secondary outcome.

##### Short Form-36 Health Survey

The Short Form-36 Health Survey [55] is a 31-item self-report measure of health-related quality of life that generates 2 scores: a mental composite score and a physical composite score. Scores are calculated using subscales of physical functioning, limitations, energy, emotional well-being, social functioning, pain, and general health. The difference in scores from the baseline to the 2-year follow-up was a secondary outcome.

##### Patient-Reported Outcomes Measurement Information System Sleep Related Impairment

The Sleep Related Impairment (version 1.0) [56] is a 16-item measure of perceived functioning during waking hours in relation to tiredness and trouble sleeping. A raw score of the items was translated into a *t* score with SE. The difference in the total score from the baseline to the 2-year follow-up was a secondary outcome.

##### Diet Self-Efficacy Scale

The Diet Self-Efficacy Scale [57] is a 12-item self-report measure of self-efficacy for healthy eating specific to physical activity, healthy eating, and weight loss. The change in the total score (sum of all items) during the initial treatment period (6 months) and follow-up period (30-month outcome) was the primary outcome.

##### Self-reported Health Status

Health status is assessed using an adapted Centers for Disease Control National Center for Health Statistics National Health Interview Survey [58]. This measure includes questions about the type and frequency of health care sought. The frequency of provider visits will be calculated to determine the use of health care from baseline to 2-year follow-up as a secondary outcome.

##### Concomitant Weight Loss Intervention Measure

The use of any weight loss resources outside the study intervention will be measured at each follow-up. Resource types could include tracking progress toward a goal, communicating with a professional, sharing weight loss experiences with others, connecting with others about a weight loss experience, and reading articles or information to learn about weight loss, healthy eating, or exercise.

#### Noom Inc Use Data

##### Adherence

Both local and global adherence will be measured during the 6 months of the intervention, which will allow the evaluation of whether program activities were implemented as intended. Local adherence will include the percentage of completed versus expected activities in each of the available features of the Noom intervention (ie, the number completed/the number expected × 100%). Features measured for the Noom Healthy Weight Program included self-monitoring of diet, physical activity, weigh-ins, engagement with group members, coach communication, and reading psychoeducational materials. The expected engagement is determined by the treatment algorithm and varies by event and week. The data were collected passively within the app. Self-monitoring of diet will be calculated as the average of daily logged meals (user input)/5 (the number of times per day that a person is expected to eat) × 100%. Global adherence will be measured using a similar calculation for completion of scheduled coach check-ins. Features measured for Noom Healthy Weight Control included self-monitoring of diet, physical activity, weigh-ins, and reading psychoeducational materials.

##### Attrition

Formal withdrawal from the study or failure to complete scheduled assessments constituted a dropout for the study.

##### Compatibility

We will capture and categorize the types of technological problems encountered by participants during the intervention and document any fixes required. The percentage of successful fixes during the first 12 months will be our compatibility measure.

##### Regular Eating

Daily regular eating will be defined by evidence of recorded 3 meals and 2 snacks within the app that were separated by >3 and <6 hours. Weekly metrics for the frequency of regular eating will be derived from these self-reported data and used in the time-series models described below.

### Data Analytic Plan and Sample Size Considerations

#### Sample Size Considerations

To determine the statistical power, we conducted Monte Carlo simulation studies [59] with 10,000 draws to estimate (1) the effect of treatment on latent growth over treatment (slope_tx_) and posttreatment follow-up (slope_FU_). We studied a model with 6 time points and 2 growth processes (slope_tx_/slope_FU_). The power to detect 1 unit difference in outcome, assuming (SD 4.5) of the treatment effect (β*_t_*_x_) on each growth process (slope_tx_, slope_FU_) was 0.92 to 94 with sample (N=600) and assuming (Cronbach α=.05). We varied patterns of missing data from 10% to 30%, increasing linearly over time, and retained >80% power to detect a 1.2-unit difference (SD 4.5) with the same sample size at 30% missing data. For within-treatment change, power with the same sample size was estimated at 90% to 98% power for a 2.5-unit change (SD 5) and a correlation of 0.5 between repeated measures. All the estimates assumed an α level of .05. Consequently, we were able to detect a difference of 1 BMI (SD 4.5) at the end of treatment and 1.2 BMI (SD 4.5) at follow-up, assuming a possibility of 10% to 30% attrition.

The power of the aim 2 moderator models involved adding latent variables based on 12 indicators. Using the above model with inclusion of an interaction term between latent moderator (M*) and treatment, we estimated 0.85 to 0.93 power to detect significant interaction assuming an effect size of 0.20 SD units for interaction above the 0.20 SD units attributable to the intervention and an α level of .05. For aim 3, power analyses were based on adherence measurements adapted from a previous project using Noom with binge eating [60] and assumed a network efficiency of 0.75. The adherence measurements included 13 data points extracted from the Noom app. We plan to examine the relationship between network entropy and outcomes, with statistical power for regression prediction at 100% for each outcome. A mediation model of weekly regular eating is a time-series mediation model and assumes that latent measures of meal-snack adherence mediate changes in weight. The calculation is based on effect size goals of 0.25, 0.50, and 0.75. The results of simulations suggest that the power for the smallest effect size (0.25) is approximately 0.80 with 10% missing data or less and moderate correlations between time and change in regular eating (0.35) and moderate correlation between regular eating and weight outcome (0.35).

#### Analysis and Statistical Methods

All available data will be used in outcome analyses. Missing data will be initially modeled as missing at random, and we will follow-up the parameter estimation of hypothesized effects with a sensitivity analysis comparing estimates to missing not at random models and imputation models.

#### Specific Aim 1

The primary and secondary end points included testing the comparative efficacy of the intervention versus control for weight loss, quality of life, psychosocial functioning, and self-reported health status. Weight loss was the primary end point, while quality of life, psychosocial functioning, and self-reported health status were secondary end points.

- Hypothesis 1: intervention users will show a significant advantage in the 6-month efficacy relative to the control for weight loss—after the intervention.
- Hypothesis 2: intervention users will show long-term (30 months) efficacy relative to the control program.

Our primary model will include 2 sequential latent growth curves modeled on the weight loss phase (0, 1, 4, and 6 months) and maintenance phase assessments (12, 18, 24, and 30 months). Latent growth curve modeling offers an advantage in terms of statistical power, as it accounts for measurement errors. In addition, this method capitalizes on the features of structural equation modeling and can examine variables simultaneously as independent and dependent variables, allowing for complex representations of growth and correlates of change [61]. The details of the model are described as follows [62]: Randomization variables (intervention vs control) are exogenous to the model and conditioned on each latent slope. The hypotheses that β_treatment_≠0 and β_maintenance_≠0 will be tested in the same model. We will test linear and nonlinear constraints on slope parameters to ensure an adequate fit and choose the best-fitting model using the Bayesian information criterion, Akaike information criterion, and root-mean square error of approximation. We will use the same modeling procedure for each of the primary outcomes.

#### Specific Aim 2

The secondary end point includes comparing potential moderators of treatment response in the intervention and control groups.

- Hypothesis 1: the clinical profile (composite moderator) will show a significant interaction with treatment condition, demonstrating that individuals with a more severe profile (older age, less support, more health conditions, and more psychosocial problems) will benefit more from the intervention than the control.
- Hypothesis 2: the difference between the lifetime highest and current weight will moderate the efficacy of the intervention or control. (Weight suppression has a larger negative impact on the control condition).

We will follow the 2-step procedure outlined by Kraemer [35] to generate a composite moderator derived from demographic and baseline severity data and participant baseline data to data reduction through principal component analysis while examining loadings to assess fit with the single severity or vulnerability profile. If evidence of multiple latent profiles exists, we will adapt our model accordingly, although our data reduction strategy will favor single-factor (eg, principal components vs geometric rotation) methods. The candidate variables include age, sex, race or ethnicity, medical conditions, medication use, and psychosocial measures included in the assessments section. Only those variables with >0.5 factor loadings will be retained for the final latent variable, and we will then incorporate the latent moderator variable into the piecewise growth models and test the interaction between the moderator and treatment. For hypothesis 2, we will use the same general approach to examine the interaction effect of the difference between the highest weight and current weight, and treatment on the proposed outcomes.

#### Specific Aim 3

This exploratory aim intends to identify the treatment mechanisms of change in Noom’s behavior change program, using a repeated-measures Bayesian belief network to establish adherence within the Noom Healthy Weight Program and examine changes over time.

- Hypothesis 1: the time-series Bayesian belief network model of adherence predicts study dropout, successful weight loss, and quality of life.
- Hypothesis 2: regular eating will mediate treatment effects of weight loss over time
- Hypothesis 3: self-efficacy of weight loss will mediate Noom Healthy Weight Program (intervention) or Noom Healthy Weight Control effects after the treatment and at the 12-month follow-up.

We will build an adherence model according to the local or global structure, as noted earlier, and test models via cross-validation on an 80/20 split of data before model building. The structural and individual parameter weights of the nodes and their stability over time will describe adherence, and the predictive accuracy will evaluate the utility of the model in practice. We will examine individual network performance metrics as mediators in a time-series estimate of causal effects in hierarchical within-participants models [63].

Exploratory hypotheses 2 and 3 will use measures of regular eating and weight loss self-efficacy during the first 3 months of treatment as mediators. Each model will use bias-corrected bootstrapped estimates of the indirect effect as our test of mediation [64].

#### Statistical Analysis: General Approach

We are using a latent growth curve model (piecewise) to model the separate change processes assumed during weight loss and after intervention [64,65]. All models will be examined for goodness of fit, normality assumptions, and linear versus nonlinear changes. Best-fit models will be interpreted for treatment effects (primary aim), and moderator models will be explicitly tested by integrating the latent variable into a best-fitting model.

With regard to baseline descriptive statistics, baseline variables (demographics and anthropometrics) will be compared using chi-square and *t* tests to determine if pretreatment bias exists in randomization. Data will be examined for robust outliers (ie, values that are not plausible, such as a BMI change of 60 kg/m^2^ between assessments), and extreme values will be monitored and validated if they exist within the range of physiology or the scale of the measures. All data cleaning and analysis will be performed without knowledge of the conditions. The principal investigator will be responsible for the analysis and will be blinded to the conditions in any study reports throughout the project. Blinding will be broken only after the analysis for the specific aim has closed. All other staff will not be blinded to the condition.

## Results

Enrollment began in March 2021, and the 6-month intervention phase ended in March 2022. Data collection for the final assessment will be completed in March 2024.

## Discussion

### Principal Outcomes

This study provides a description of the rationale, design, and procedures for testing the efficacy and mechanisms of change in a digital health intervention for individuals with overweight and obesity. We anticipate that overall benefits, and specific improvements for individuals in the higher-risk profile will be observed after the intervention in the Noom Healthy Weight Program compared with Noom Healthy Weight Control. The design allows an examination of 6 months of intervention and 24 months of maintenance outcomes to answer a fundamental question about the long-term value of digital weight loss interventions. To test the hypothesized long-term efficacy of the Noom Healthy Weight Program (intervention), the Noom Healthy Weight Control (control) retains the same digital format, access, and fundamental components of behavior change, with the exception of prescribed weekly advice delivered via articles rather than by a Noom coach, availability for group communication, and limited logging features. Consequently, inferences about intervention effects will be more closely linked to the mechanistic hypotheses embedded in the intervention and control designs.

### Expected Novel Contributions From the Research

Adherence to weight loss interventions is an important factor to consider when evaluating the efficacy of an intervention. There is limited knowledge about the level of adherence needed for successful weight loss and few standard measures of adherence [22,66,67]. Adherence is difficult to track with the complex elements of a lifestyle intervention such as didactic information delivery, level and frequency of daily monitoring, length and quality of coach contact, and the participant’s choice of meaningful changes. Smartphones provide a greater opportunity to model individual variability in specific behaviors prescribed by lifestyle interventions. Tracking how users interact with each feature in the program allows for the ability to measure use (eg, frequency of logging) and the application of intervention lessons (eg, Did coach interactions indicate that the participant was using intervention components?). Furthermore, self-efficacy plays a critical role in weight loss, as the way an individual thinks, behaves, and perceives success contributes to improved weight loss, better health choices [68], and confidence in making healthy decisions. The ability to successfully solve weight loss barriers contributes to successful long-term weight loss maintenance [69,70] and better adherence to intervention protocols [71]. This project will allow us to parse out the effects of the Noom Healthy Weight Program on adherence, self-efficacy, and regular eating from the common general effects of digitized behavioral weight loss such as support, goal setting, and logging [16].

In addition, this design introduces a new approach to studying moderator effects, aiming to profile individuals by demographic and severity indicators at baseline, allowing for the collective sum of smaller potential moderators to contribute to potentially important and clinically meaningful differences in response to these treatments.

Limited information exists regarding the long-term effectiveness of digitally delivered behavioral weight loss programs [24]. Weight loss maintenance has been achieved through in-person programs using continued access to coaches and logging [19,30]. However, in-person treatment is often too costly or uncovered by insurance and remains inaccessible for many people [30,72]. Participants providing feedback in other weight loss trials reported that weight loss success would be greater if access is provided to these intervention components for long term and at no cost or low cost [20,73]. Mobile apps provide a viable and cost-effective alternative. Testing the logging, coaching, and group features of Noom over the long term will provide important information about the association between the use of digitally delivered behavioral strategies and maintenance of weight change.

### Alternative Design Considerations

We considered alternative digital platforms for comparison with our intervention condition; however, the constant evolution of commercial and noncommercial alternatives increased the risk that interventions could be contaminated in the case of a substantial change in delivery. Furthermore, using 2 different versions of Noom Inc will ensure a similar user experience with visual presentation, design, and functionality, thus reducing the possibility of group differences resulting from differences between programs. Recruiting users directly from Noom Inc allows us to target individuals interested in using digital interventions for weight loss and users motivated to lose weight. Local sampling (eg, from hospital programs or local environments) and stratified sampling strategies were considered but posed threats to generalizability. Selecting individuals seeking intervention from Noom Inc allows us to provide the most robust test of the existing platform, which has adapted the experience with its consumer base. Therefore, our national recruitment kept our sampling as wide as possible by enrolling those who focused on general app weight loss and minimalizing exclusion criteria. However, we recognize that the choice of a non–coach control condition limits full control of the active ingredients of the experimental condition and generalizability of the effects to Noom Inc’s noncoaching products.

### Limitations

The limitations of this study include potential for intervention contamination and self-directed weighing. It is possible that study participants could use weight loss resources outside of what is specifically delivered via the study intervention; however, we will be able to measure concomitant treatment use at study follow-up as a potential confounder. Owing to feasibility, timeline, and cost, weight will not be measured via a standard scale or with scales that have similar specifications.

### Conclusions

Our study will use the technology of the Noom platform to overcome obstacles to weight loss and long-term weight loss maintenance, such as accessibility and the use of digitally delivered evidence-based features. In addition, this platform provides us with the opportunity to measure adherence to a digitally delivered behavioral intervention while exploring the role of each feature.

## Abbreviations

DPP: Diabetes Prevention Program

## Data Availability

At the end of the trial, results will be posted on ClinicalTrials.gov, and data that support the findings of this study will be available from the corresponding author (RS) upon reasonable request.
